# Longitudinal Effects of the Home Learning Environment and Parental Difficulties on Reading and Math Development Across Grades 1–9

**DOI:** 10.3389/fpsyg.2020.577981

**Published:** 2020-10-08

**Authors:** Daria Khanolainen, Maria Psyridou, Gintautas Silinskas, Marja-Kristiina Lerkkanen, Pekka Niemi, Anna-Maija Poikkeus, Minna Torppa

**Affiliations:** ^1^Department of Teacher Education, University of Jyväskylä, Jyväskylä, Finland; ^2^Department of Psychology, University of Jyväskylä, Jyväskylä, Finland; ^3^Norwegian Centre for Learning Environment, University of Stavanger, Stavanger, Norway; ^4^Department of Psychology, University of Turku, Turku, Finland

**Keywords:** reading difficulties, mathematical difficulties, home literacy environment, home numeracy environment, familial risk, skill development, comorbidity

## Abstract

This study focuses on parental reading and mathematical difficulties, the home literacy environment, and the home numeracy environment as well as their predictive role in Finnish children’s reading and mathematical development through Grades 1–9. We examined if parental reading and mathematical difficulties directly predict children’s academic performance and/or if they are mediated by the home learning environment. Mothers (*n* = 1590) and fathers (*n* = 1507) reported on their reading and mathematical difficulties as well as on the home environment (shared reading, teaching literacy, and numeracy) when their children were in kindergarten. Tests for reading fluency, reading comprehension, and arithmetic fluency were administered to children in Grades 1, 2, 3, 4, 7, and 9. Parental reading difficulties predicted children’s reading fluency, whereas parental mathematical difficulties predicted their reading comprehension and arithmetic fluency. Familial risk was associated with neither formal nor informal home environment factors, whereas maternal education had a significant relationship with both, with higher levels of education among mothers predicting less time spent on teaching activities and more time spent on shared reading. In addition, shared reading was significantly associated with the development of reading comprehension up to Grades 3 and 4, whereas other components of the home learning environment were not associated with any assessed skills. Our study highlights that taken together, familial risk, parental education, and the home learning environment form a complex pattern of associations with children’s mathematical and reading skills.

## Introduction

Literacy and numeracy development are strongly interrelated, and the comorbidity of reading and mathematical difficulties is frequent (e.g., Purpura et al., 2011; Davidse et al., 2014; Purpura and Ganley, 2014; Korpipää, 2020). Of the people with either reading or mathematical difficulties, up to 70% also perform worse than average in the other domain (Landerl and Moll, 2010; Moll et al., 2019; Joyner and Wagner, 2020). Research has identified multiple shared and unique risk factors for reading and mathematical difficulties at the level of cognitive skills (Geary, 2011; Moll et al., 2016; Child et al., 2019) and brain processes (Raschle et al., 2011; Evans et al., 2015; Norton et al., 2015). At the etiological level, both reading and mathematical difficulties are known to be heritable (Kovas et al., 2013; de Zeeuw et al., 2015; Little et al., 2017). Having a parent with reading difficulties, for example, increases the risk of children developing similar problems by up to 66% (van Bergen et al., 2014a; Hulme et al., 2015; Torppa et al., 2015; Esmaeeli et al., 2019). Significantly less is known about familial risk (FR) for mathematical difficulties (e.g., Soares et al., 2018). FR acts via genes, but environmental factors have been shown to play an important role in the development of both reading (Evans and Shaw, 2008; Mol and Bus, 2011; Manolitsis et al., 2013) and mathematical skills (Dunst et al., 2017; Daucourt, 2019). Studies on the interaction of FR and the home literacy environment (HLE) are emerging (Hamilton et al., 2016; Dilnot et al., 2017; Esmaeeli et al., 2018), but comparable studies on the home numeracy environment (HNE) remain scant (Silinskas et al., 2010). Moreover, until recently, HLE and HNE have been separately studied, whereas their cross-domain and joint roles in children’s reading and mathematical development have received very little research attention.

In view of the existing gaps in the literature, this study aims to gain new insights into the etiology of the comorbidity of reading and mathematical difficulties. To this end, the study examines the effects of FR for mathematical and reading difficulties together with the effects of the HLE and HNE on children’s (aged 7–16 years) reading and mathematical skills from a long-term developmental perspective. To our knowledge, this is the first study with such an objective.

### Familial Risk and the Comorbidity of Reading and Mathematical Difficulties

The multiple deficit model (e.g., Pennington, 2006) explains the emergence of learning difficulties and their comorbidity by the complex interactions between multiple risk factors at different levels (genes, brain, cognition, and environment), which can be either domain-specific (i.e., associated only with difficulties in one domain—either reading or mathematics) or domain-general (i.e., associated with difficulties in multiple domains). It has been established that, for example, a deficit in phonological awareness is specific to reading difficulties (Melby-Lervåg et al., 2012) and a deficit in numerosity processing is specific to mathematical difficulties (Hannula et al., 2010; Anobile et al., 2016), whereas difficulties in working memory, processing speed, and oral language are likely to affect more than one learning domain (Koponen et al., 2007; Moll et al., 2019; Daucourt et al., 2020).

The multiple deficit model (MDM) has gained wide recognition over the years. However, Pennington (2006) importantly noted that compared with single deficit models, testing the MDM would represent a much more serious challenge, calling for the test of multiple hypotheses. In their theoretical article, van Bergen et al. (2014b) stressed the unique role of familial risk studies in testing and specifying the MDM—these studies have already provided important evidence suggesting that parents confer liability to reading difficulties via interconnected genetic and environmental risk factors.

In this study, we aim to add knowledge on the intergenerational transmission of reading and mathematical difficulties as well as their comorbidity. To this end, we include FR for both reading and mathematics and examine the effects of both within-domain and cross-domain FR on reading and mathematical development. Although multiple studies have established that FR for reading difficulties is among the strongest predictors for dyslexia (Scarborough, 1990; Pennington and Lefly, 2001; van Bergen et al., 2014a; Torppa et al., 2015; Esmaeeli et al., 2019), so far, only few studies have suggested that the same is true for dyscalculia (Shalev and Gross-Tsur, 2001; Soares et al., 2018). In addition, unlike most studies, we include the parental reading and mathematical difficulties of both mothers and fathers in our analysis to examine if the effects of having one parent with difficulties are different from the effects of having both parents with difficulties. Based on the MDM, it can be expected that when both parents have learning difficulties, children’s liability increases more than when having only one parent with difficulties.

### Home Literacy and Numeracy Environment

The effects of FR on children’s skill development may act through the genetic pathway; both twin and molecular genetic studies have produced compelling evidence for the strong heritability of both reading and mathematical skills (Docherty et al., 2010; Kovas et al., 2013; de Zeeuw et al., 2015; Little et al., 2017). However, parental reading/mathematical difficulties have also been shown to be transmitted through the environmental pathway (Petrill et al., 2005; de Zeeuw et al., 2015; Hart et al., 2016; van Bergen et al., 2017). Therefore, we examine if parental reading and mathematical difficulties impact the home environment and if they affect children’s skills not only directly but also indirectly via the home environment.

The home learning environment is often divided into two main components: HLE and HNE. HLE refers to home-based interactions between parents and their children, parental attitudes, and at-home materials related to literacy. HLE has long been considered an important factor for the development of reading skills (see Bus et al., 1995; Evans and Shaw, 2008; Flack et al., 2018; Grolig et al., 2019). In a seminal study, Sénéchal and Lefevre (2002) formulated the home literacy model and showed that to adequately assess the effects of HLE, it is important to differentiate its activities into two separate categories: “formal” and “informal” activities. In their 5-year longitudinal study, children’s skills were followed until the end of Grade 3 and HLE was assessed with parental self-reports. The home literacy model was predicated on analysis that revealed that parental teaching (formal learning) and storybook exposure (informal learning) were uncorrelated, with the former explaining children’s emergent literacy and the latter explaining children’s receptive language.

Further evidence has supported the home literacy model, showing that formal and informal activities contribute to the development of different skills (Sénéchal and Lefevre, 2002). Code-related, formal parent–child literacy interactions in the form of direct teaching (for example, instructing children on how to divide words into phonemes and showing that graphemes correspond to phonemes) contribute to the development of early word recognition and decoding skills, whereas informal literacy activities (for example, shared reading and discussions over a story) mostly involve meaning-related practices and are associated with the development of vocabulary knowledge, reading comprehension, and broader language skills (e.g., Sénéchal, 2006, 2015; Mol et al., 2008; Sénéchal et al., 2008; Martini and Sénéchal, 2012; Sénéchal and Lefevre, 2014).

However, some studies have reported negligible independent effects of formal and informal HLE activities. For example, Manolitsis et al. (2013) and Silinskas et al. (2020) found that the effects of formal learning (at-home teaching) were significantly smaller in the contexts of transparent orthographies (Greek and Finnish) than those previously demonstrated in the contexts of opaque orthographies (English and French). The authors argued that in the context of transparent orthographies, direct at-home teaching could only provide short-term gains that fade away as soon as children get exposed to schooling because learning to read is relatively easy and most children very quickly learn to read.

Using the home literacy model (Sénéchal and Lefevre, 2002) as a guiding framework, a similar model for HNE was developed and tested by Skwarchuk et al. (2014). In a cross-sectional study with 5- and 6-year-old children, the researchers assessed the formal activities of HNE (using parental self-reports of home teaching of arithmetic skills) and informal activities (using a number game title checklist for parents, which is comparable to the storybook exposure checklist designed for HLE). The study revealed that formal parent–child interactions contributed to children’s symbolic number knowledge (number identification, counting, and ordinal numbers), whereas informal game-based numeracy-related activities contributed to children’s non-symbolic arithmetic skills (addition, subtraction, and matching tasks with toy animals).

It has to be stressed, however, that research focusing on the role of HNE remains rather scant and much less conclusive in comparison to studies on HLE. Whereas some studies suggest that the HNE is a significant contributor to the development of mathematical skills (Niklas and Schneider, 2014; Skwarchuk et al., 2014; Hart et al., 2016; Napoli and Purpura, 2018), other research finds a non-significant or even negative association between children’s mathematical development and HNE (Blevins-Knabe et al., 2000; Silinskas et al., 2010; Missall et al., 2015; Zippert and Rittle-Johnson, 2020).

Importantly, from the perspective of understanding comorbidity, a recent study among parents of children aged 3–5 years (Napoli and Purpura, 2018) established a strong relationship between HLE and HNE after analyzing extensive parental self-reports of at-home literacy practices (printing letters, identifying letters and letter sounds, and reading storybooks) and numeracy practices (counting objects, printing numbers, working with number activity books, comparing quantities, counting down, and learning written numbers and simple sums). Results showed that the parents who were actively promoting the skills of their children in one domain were more likely to do the same in the other domain (Napoli and Purpura, 2018). This strong positive association between HLE and HNE could be one of the reasons why researchers find that HLE predicts both reading and mathematical skills (Melhuish et al., 2008; Baker, 2014). In a longitudinal study with pre-school children aged 3–4 years who were followed for 3 years, Anders et al. (2012) found that HLE was an even better predictor of early mathematical skills than HNE. The researchers argued that verbal literacy is a pre-requisite for acquiring numeracy skills, as has been suggested by von Aster and Shalev (2007) and later reported by Purpura and Ganley (2014). This evidence shows that studying both HLE and HNE together is necessary to understand the impact of the home environment on children’s skill development. Noting that previous studies mainly focused on early childhood, the present study aims to add knowledge on how the processes of developing reading and mathematical skills are interconnected by extending research to school-aged children. Furthermore, the inclusion of FR and parental education in our study enables us to investigate if the possible correlation between HLE and HNE can be further explained to help understand why some parents are more likely to support their children’s skill development (Napoli and Purpura, 2018).

### Familial Risk Studies and Home Learning Environment

To establish whether FR is mediated via the home learning environment, studies have compared the HLE factors in families with and without FR for reading difficulties. Whether such an indirect relationship exists, however, is still unclear owing to the scarcity of research (e.g., Snowling and Melby-Lervåg, 2016) as well as to contradictory findings. Some studies found that FR families provide a more disadvantageous HLE for their children than non-FR families do (Hamilton et al., 2016; Dilnot et al., 2017; Esmaeeli et al., 2018). Other studies reported that there were no significant differences between the at-home learning activities of FR families and non-FR families and that parents with reading difficulties taught their children as much academic skills as the parents without such difficulties did (Elbro et al., 1998; Laakso et al., 1999; Torppa et al., 2007). Comparable studies investigating FR for mathematical skills and HNE are scarce. However, in one longitudinal study, Silinskas et al. (2010) showed that Finnish mothers’ mathematical difficulties positively predicted their teaching of mathematics.

Few studies have gone further to investigate if HLE can act as a mediator between parental reading difficulties and children’s literacy outcomes. In their large-scale study with 6-year-old children, Esmaeeli et al. (2019) suggested that HLE could play the role of a protective factor mediating the adverse influences of FR on children’s reading skills. However, Puglisi et al. (2017) reported that informal HLE did not predict any children’s outcomes when maternal language and phonological skills were controlled for. The researchers then argued that the associations found between children’s skills and informal HLE might only be a reflection of intergenerational transmission—parents with stronger language skills involve their children in more informal learning activities but also provide genes that predispose their children to have stronger language skills. To disentangle these familial and environmental influences, more studies are needed.

To summarize the previous research, numeracy and literacy are highly interconnected, complex cognitive skills and parents can pass down both reading and mathematical difficulties to their children through genetic and environmental pathways. The exact mechanism of a child developing either one or both sets of difficulties remains poorly understood, but it appears that this process is shaped by the interaction of multiple deficits (domain-specific and domain-general). Moreover, HLE has been repeatedly shown to be associated with children’s language and literacy development, and in some recent studies also with mathematical skill development. Clear effects of different HNE activities on numeracy have been found only in a handful of studies and require more research. There is also a particular need for more studies on FR for mathematical difficulties, cross-domain FR effects, and parental comorbidity effects on the development of reading and mathematical skills. In addition, it remains to be seen if FR and non-FR families provide different HLE and/or HNE, and if the influence of FR on children’s skills can be mediated through the home environment.

### Present Study

Our analysis of the gaps in research suggests that further exploring how the development of reading and mathematical skills is influenced by parental reading and mathematical difficulties (FR for reading and mathematics, respectively) as well as home environment factors is important. Evidence from previous studies is scant because most of the studies on HLE and HNE were cross-sectional and/or small-scale and focused on early development. In contrast, the present study is a large-scale longitudinal study spanning across the compulsory education until adolescence. Based on theory and previous empirical evidence, we divided environment variables into formal (teaching of literacy and numeracy skills) and informal home inputs (shared reading) (Sénéchal, 2006; Sénéchal and Lefevre, 2014; Hamilton et al., 2016; Puglisi et al., 2017). Because parental education has been shown to be reflected in HLE (e.g., Torppa et al., 2006; Park, 2008; Hamilton et al., 2016; van Bergen et al., 2017), it is included in all our models.

We aim to answer the following research questions:

(1) Does FR for reading and/or mathematical difficulties predict the reading and mathematical development of children from Grade 1 to 9?
(2) Do home environment factors (literacy teaching, numeracy teaching, and shared reading) predict the reading and mathematical development of children from Grades 1–9?
(3) Does FR for reading and mathematical difficulties predict the home learning environment?
(4) Are the effects of FR on children’s reading and mathematical development mediated by the home environment factors?

**FIGURE 1:**
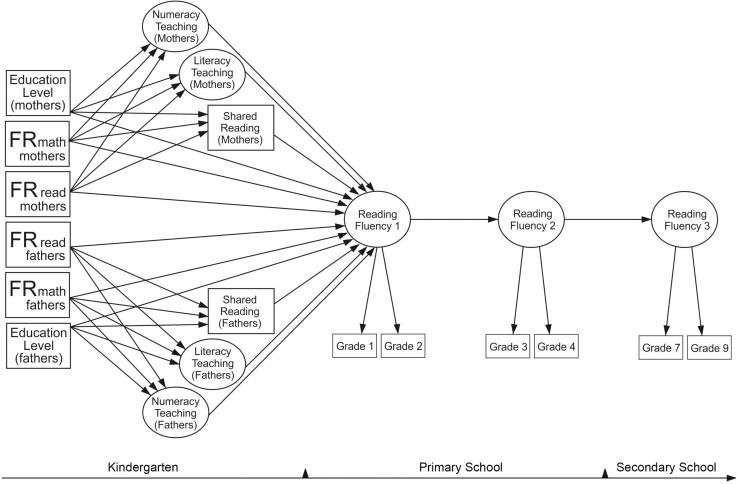
Hypothesized model for reading fluency. Familial risk (FR for reading and mathematical difficulties of mothers and fathers); Reading Fluency 1–Reading Fluency 3, time points of assessments; Grade 1–9, assessments that took place in Grades 1–9. Literacy teaching and numeracy teaching were added in the model as latent variables; they were measured with two questionnaire items each. Shared reading was measured with one questionnaire item making up the observed variables (one for mothers and one for fathers). Other hypothesized models (for reading comprehension and mathematical skills) were constructed with the same logic.

In this study, we estimate three different models: for reading fluency, for reading comprehension, and for arithmetic fluency based on our hypothesized models. In view of the research reviewed above, we constructed our hypothesized models (see Figure 1 for the model of reading fluency; other models were estimated with the same logic) with the expectation to find the following: (1) paths from parental reading difficulties (Pennington and Lefly, 2001; Torppa et al., 2011; van Bergen et al., 2012; Hulme et al., 2015) and parental mathematical difficulties (Shalev and Gross-Tsur, 2001; Soares et al., 2018) to the respective skills in children; (2) cross-domain paths from parental mathematical difficulties to children’s reading skills and from parental reading difficulties to children’s mathematical skills (Landerl and Moll, 2010; Moll et al., 2015); (3) paths from HLE and HNE to both respective and cross-domain skills in children (Melhuish et al., 2008; Anders et al., 2012; Kleemans et al., 2012; Baker, 2014; Napoli and Purpura, 2018); (4) paths from parental education to children’s skills (Torppa et al., 2006; Hamilton et al., 2016; van Bergen et al., 2017); (5) paths from parental education to HLE and HNE (Hamilton et al., 2016); and (6) paths from FR to the home environment (Scarborough et al., 1991; Bus et al., 1995; Elbro et al., 1998; Snowling, 2000; Hamilton et al., 2016; Esmaeeli et al., 2019), including also the examination of the indirect relationships (FR → home environment → children’s skills), as Esmaeeli et al. (2019) argued that these paths need to be tested in future studies. Finally, we expected that the paths to later skill assessments run through the early skill assessments.

## Materials and Methods

### Participants and Procedure

This study is a part of a large-scale longitudinal First Steps Study (Lerkkanen et al., 2006) where children (*n* = 2525) were followed from kindergarten to Grade 9. The children were born in the year 2000 and came from four municipalities: one in an urban area, one in a rural area, and two in, similarly, semi-rural areas in central, western, and eastern Finland. Of all contacted families, 78–89%, depending on municipality, agreed to participate in the study. Ethnically and culturally, the sample was very homogeneous and representative of the Finnish population. Marital statuses as well as the educational levels of the parents were very close to the national distribution of Finland (Statistics Finland, 2007). The study was reviewed and approved by the Ethical Committee of the University of Jyväskylä in 2006, and all participants (children and their parents) gave their informed consent before participation in the study.

Trained specialists administered both individual and group tests in suitable rooms in each school. Children absent from school on the day of testing were tested immediately after they came back to school. Tests for reading fluency, reading comprehension, and mathematics were administered to children in Grades 1, 2, 3, 4, 7, and 9.

### Measures

#### Reading Fluency

To assess reading fluency, three group-administered tests were administered: a word reading fluency task, a word chain task, and a sentence reading task. The mean of the three standardized reading fluency measures was used as the score. Cronbach’s alpha reliability coefficients for the fluency composite were 0.94 in Grade 1, 0.93 in Grade 2, 0.93 in Grade 3, 0.93 in Grade 4, 0.93 in Grade 7, and 0.94 in Grade 9.

The word reading fluency task is an 80-item subtest of the nationally normed reading test battery (ALLU; Lindeman, 2000). Each item comprises a picture and a set of four phonologically similar words. The children were asked to silently read the words and decide which one of them semantically matched the picture. All the words and pictures in the task were simple and frequently used and thus were familiar to young children. The score was calculated as the number of correct answers achieved within 2 min. The score reflects both the word-reading speed and accuracy.

In the word chain task (Nevala and Lyytinen, 2000), children were presented with 10 chains of 4–6 words in a row written without spaces between them. The children were asked to silently read each row and draw a boundary line between each word pair they find. The sum score was based on the number of correct answers given within a set time limit (1.25 min in Grades 1 and 2, 1.20 min in Grade 3, 1.05 min in Grade 4, 1 min in Grades 6 and 7, and 1.30 min in Grade 9).

Sentence reading efficiency in Grades 1–4 was assessed with the Test of Silent Reading Efficiency and Comprehension (TOSREC; Wagner et al., 2010; Finnish version by Lerkkanen and Poikkeus, 2009). The children were asked to read and assess the truthfulness of as many simple sentences as possible (e.g., Strawberries are blue) out of a set of 60 items within 3 min. In Grades 7 and 9, the children were asked to complete a standardized Finnish reading test for lower secondary school sentence reading that had the same instruction as earlier sentence reading measures but slightly different items (YKÄ; Lerkkanen et al., 2018) were used. The sum score was based on the number of correct answers.

#### Reading Comprehension

To assess reading comprehension in Grades 1–4, a group-administered subtest of a nationally normed reading test battery was used (ALLU; Lindeman, 2000). The children were required to read a short fiction story and answer 11 multiple-choice questions and 1 question in which they had to arrange 5 statements in the correct sequence based on the information gathered from the text. For each correct answer, 1 point was given (max = 12). The children could work at their own pace but for a maximum of 45 min. Then, in Grades 7 and 9, a similar standardized reading comprehension test for lower secondary school (with the same instruction and time limit but different texts and questions) was employed (YKÄ; Lerkkanen et al., 2018). The sum score was based on the number of correct answers. Cronbach’s alpha reliability coefficient for the comprehension composite ranged between 0.82 and 0.84 in different grades (0.84 in Grade 1, 0.82 in Grade 2, 0.83 in Grade 3, 0.82 in Grade 4, 0.82 in Grade 7, and 0.83 in Grade 9).

#### Arithmetic Fluency

Arithmetic fluency was assessed with a group-administered subtest of the arithmetic test (Räsänen and Aunola, 2007) that comprises 14 addition (e.g., 3 + 2 = __, 3 + 6 + 4 = __) and 14 subtraction tasks (e.g., 6 − 1 = __, 20 − 4 − 3 = __). Performance on this test depends on both speed and accuracy, and allows for the assessment of the automatization of basic mathematical computations. The sum score was based on the number of correct answers given within 3 min. Cronbach’s alphas varied between 0.91 and 0.92 (0.92 in Grade 1, 0.91 in Grades 2–4, 7, and 9).

#### Familial Risk for Reading Difficulties

When the children participating in the study were in kindergarten, their mothers and fathers were asked to fill in a questionnaire asking if they themselves and/or the other parent of the child had experienced learning difficulties in reading and/or mathematics. The questionnaire included one question about their own reading difficulties, one about their own mathematical difficulties, and two in regard to their spouse. Each question could be answered on a three-point scale (1 = no difficulties, 2 = some difficulties, 3 = clear difficulties). The children were considered to have FR if they had at least one parent with some or clear difficulties, and the variable for FR was then dichotomized: 0 = no FR (report of no difficulties) and 1 = FR (report of some or clear difficulties). In the descriptive analysis, we also considered if a child has one or two parents with learning difficulties (Tables 2, 3).

#### Parental Education

Mothers and fathers were asked to indicate their own educational level on a seven-point scale [1 = no vocational education (5.1% of mothers and 1.8% of fathers), 2 = vocational courses (3.1% of mothers and 1.7% of fathers), 3 = vocational school degree (30.8% of mothers and 14.3% of fathers), 4 = vocational college degree (23.2% of mothers and 10.1% of fathers), 5 = polytechnic degree or bachelor’s degree (9.7% of mothers and 4.2% of fathers), 6 = master’s degree (23.7% of mothers and 8.0% of fathers), 7 = licentiate or doctoral degree (4.4% of mothers and 2.7% of fathers)].

#### Home Learning Environment (Home Teaching and Shared Reading)

Mothers and fathers were also asked to complete a questionnaire about their at-home learning activities, which was based on the questions developed by Sénéchal et al. (1998) and previously used in the Finnish context (e.g., Silinskas et al., 2012, 2020). The questionnaire included one question regarding shared reading—“How often do you read books to your child or together with your child”? The answers were given on a five-point Likert-type scale (1 = less than once a week, 2 = 1–3 times a week, 3 = 4–6 times a week, 4 = once a day, 5 = more than once a day). There were four items related to home teaching activities: teaching letters, teaching reading, teaching numbers, and teaching arithmetic skills. The answers were given on a five-point scale (1 = never at all/rarely to 5 = very often/daily). We obtained the sum scores by summarizing the individual scores for each activity of mothers and fathers.

### Statistical Analysis

When investigating the predictive longitudinal relations between FR, home activities, and children’s skills, longitudinal path models were constructed using MPlus Version 7.4. Three separate models (Figure 1) were fitted to the data: for reading fluency, for reading comprehension, and for arithmetic fluency. Latent variables were built for reading fluency, reading comprehension, and arithmetic fluency to increase the reliability of the assessment and to minimize measurement error. The skill assessments in Grades 1 and 2 were grouped into Time Point 1, in Grades 3 and 4 into Time Point 2, and in Grades 7 and 9 into Time Point 3.

Latent factors were also built for the home environment measures. The factor structure of the home environment (shared reading and the four teaching items) was validated with confirmatory factor analysis (CFA). We first tested a model with four latent variables grouped as follows: the three literacy items of mothers (including shared reading), the two numeracy items of mothers, the three literacy items of fathers, and the two numeracy items of fathers, as it seemed theoretically plausible. However, this model had a poor fit with the data [χ^2^ (29) = 141.19, *p* < 0.001, root-mean-square error of approximation (RMSEA) = 0.05, comparative fit index (CFI) = 0.87, standardized root-mean-square residual (SRMR) = 0.07]. The main reason for the misfit was that the correlations between the literacy teaching and numeracy teaching items were too high to form separate constructs. In view of this, we next constructed a two-factor model wherein all home environment items of mothers were loaded to one factor and all home environment items of fathers were loaded to another factor. This model also did not fit the data well [χ^2^(33) = 107.31, *p* < 0.001, RMSEA = 0.04, CFI = 0.91, SRMR = 0.07]. Because the shared reading items had very low factor loadings, we constructed another model with one latent factor for mothers’ teaching items, including two items of teaching reading and two items of teaching mathematics, and another latent factor for fathers’ teaching items. Shared reading items of mothers and fathers were separately added as observed variables. This model fitted the data well [χ^2^(31) = 55.81, *p* < 0.01, RMSEA = 0.02, CFI = 0.97, SRMR = 0.03] and significantly better than the model where the shared reading item was included in the latent factor, as suggested by the Satorra-Bentler corrected chi-square difference test: Δχ^2^(1) = 22.23, *p* < 0.001. This confirmed our initial hypothesis that the shared reading items should be added in the models as separate variables (informal home environment inputs) from the teaching items (formal home environment inputs).

The measure distributions were close to normal distribution, except for comprehension in early grades that had a slight skew to the left (Table 1). Therefore, all models were estimated using Maximum likelihood estimation with robust standard errors. The variables were standardized before fitting the models. A few outliers were present in the distributions of all skills, which were moved to the tails of the distributions before analyses.

**TABLE 1:** Descriptive statistics for all variables across time.

	N	Minimum	Maximum	Mean	SD	Skewness	Kurtosis
**Reading fluency (z-scores)**							
Grade 1	2,052	−2.44	4.03	0.00	1.00	0.62	0.44
Grade 2	2,006	−2.89	3.88	0.00	1.00	0.26	0.23
Grade 3	1,995	−4.41	3.18	0.00	1.00	−0.04	0.43
Grade 4	1,954	−4.62	2.76	0.00	1.00	−0.17	−0.30
Grade 7	1,770	−4.19	3.04	0.00	1.00	−0.07	−0.00
Grade 9	1,721	−2.94	2.98	0.00	1.00	−0.09	−0.14
**Reading comprehension**							
Grade 1	2,035	0.00	12.00	5.50	3.18	−0.00	−0.96
Grade 2	1,974	0.00	12.00	8.51	2.71	−0.73	0.20
Grade 3	1,988	0.00	12.00	9.08	2.16	−1.17	1.73
Grade 4	1,950	0.00	12.00	8.10	2.52	−0.47	−0.21
Grade 7	1,758	0.00	12.00	6.59	2.54	0.05	−0.65
Grade 9	1,702	0.00	12.00	7.01	2.43	−0.15	−0.58
**Arithmetic fluency**							
Grade 1	2,050	0	28	10.51	4.12	0.33	0.25
Grade 2	2,001	0	28	16.05	4.92	−0.10	−0.45
Grade 3	1,994	0	28	19.61	4.62	−0.65	0.48
Grade 4	1,953	0	27	17.03	4.09	−0.64	0.81
Grade 7	1,749	0	27	13.68	3.81	−0.17	0.34
Grade 9	1,705	1	27	14.89	3.92	−0.13	0.05
**Parental education**							
Mother	1,563	1	7	4.18	1.52	−0.00	−0.12
Father	1,117	1	7	4.12	1.50	−0.20	−0.15
**Home learning environment factors (mean composites)**							
Shared reading, mother	1,559	1	7	2.29	1.15	−0.15	−1.01
Shared reading, father	1,104	1	7	2.35	1.15	0.47	−0.89
Teaching, mother	1,115	1	5	2.54	0.75	0.08	−0.11
Teaching, father	1,567	1	5	2.60	0.79	0.02	−0.19

**TABLE 2:** ANOVA comparisons among the three risk groups for reading difficulties (RD) for all variables.

	No family risk for RD (NFR)			One parent risk for RD (FR1)			Both parents risk for RD (FR2)					
	*N*	*M*	*SD*	*N*	*M*	*SD*	*N*	*M*	*SD*	*df* within groups	F	Pairwise comparisons (Bonferroni)
**Reading fluency (z-scores)**												
Grade 1	979	0.18	0.85	377	–0.14	0.82	58	–0.56	0.69	1,411	26.90***	NFR > FR1, FR1 > FR2, NFR > FR2
Grade 2	957	0.20	0.83	362	–0.20	0.83	58	–0.52	0.69	1,374	34.23***	NFR > FR1, FR1 = FR2, NFR > FR2
Grade 3	941	0.17	0.82	362	–0.11	0.85	57	–0.56	0.63	1,357	19.50***	NFR > FR1, FR1 = FR2, NFR > FR2
Grade 4	921	0.19	0.81	356	–0.11	0.87	53	–0.56	0.64	1,327	25.38***	NFR > FR1, FR1 > FR2, NFR > FR2
Grade 7	697	0.19	0.83	268	–0.13	0.94	33	–0.26	0.79	995	12.26***	NFR > FR1, FR1 = FR2, NFR > FR2
Grade 9	682	0.19	0.84	260	–0.07	0.91	33	–0.26	0.70	972	9.20***	NFR > FR1, FR1 = FR2, NFR > FR2
**Reading comprehension**												
Grade 1	977	6.06	3.19	373	5.13	3.08	58	4.09	2.87	1,405	20.14***	NFR > FR1, FR1 = FR2, NFR > FR2
Grade 2	945	8.98	2.51	358	8.22	2.75	58	7.50	2.93	1,358	17.81***	NFR > FR1, FR1 = FR2, NFR > FR2
Grade 3	939	9.43	1.97	361	8.79	2.29	57	8.89	2.12	1,354	13.58***	NFR > FR1, FR1 = FR2, NFR > FR2
Grade 4	920	8.58	2.29	356	7.92	2.57	53	7.58	2.54	1,326	12.77***	NFR > FR1, FR1 = FR2, NFR > FR2
Grade 7	691	7.02	2.52	268	6.51	2.63	33	5.97	2.36	989	5.88**	NFR > FR1, FR1 = FR2, NFR > FR2
Grade 9	680	7.40	2.41	255	6.96	2.39	32	6.22	1.93	964	6.20**	NFR > FR1, FR1 = FR2, NFR > FR2
**Arithmetic fluency**												
Grade 1	979	11.10	4.10	376	10.24	4.11	58	9.71	3.97	1,410	8.13***	NFR > FR1, FR1 = FR2, NFR > FR2
Grade 2	953	16.81	4.78	362	15.99	4.83	58	14.19	4.97	1,370	10.70***	NFR > FR1, FR1 = FR2, NFR > FR2
Grade 3	941	20.23	4.37	362	19.50	4.62	57	18.11	4.94	1,357	8.59***	NFR > FR1, FR1 = FR2, NFR > FR2
Grade 4	920	17.59	3.86	356	16.96	4.14	53	16.40	4.22	1,326	4.89**	NFR > FR1, FR1 = FR2, NFR > FR2
Grade 7	690	14.15	3.82	265	13.91	3.66	34	13.29	3.61	986	1.11	NFR = FR1, FR1 = FR2, NFR = FR2
Grade 9	676	15.49	3.74	256	14.70	3.87	34	14.53	3.83	963	4.69**	NFR > FR1, FR1 = FR2, NFR > FR2
**Parental education**												
Mother	1,009	4.37	1.48	397	4.04	1.48	66	3.38	1.24	1,469	19.20***	NFR > FR1, FR1 > FR2, NFR > FR2
Father	759	4.28	1.49	287	3.82	1.52	48	3.71	1.23	1,091	12.06***	NFR > FR1, FR1 = FR2, NFR > FR2
**Home learning environment factors (mean composites)**												
Shared reading, mother	1,007	2.96	1.13	397	2.86	1.16	66	2.67	1.17	1,467	2.87	NFR = FR1, FR1 = FR2, NFR = FR2
Shared reading, father	752	2.38	1.16	280	2.30	1.15	47	2.30	1.16	1,076	0.56	NFR = FR1, FR1 = FR2, NFR = FR2
Teaching, mother	1,010	2.60	0.79	399	2.59	0.79	67	2.46	0.84	1,473	0.95	NFR = FR1, FR1 = FR2, NFR = FR2
Teaching, father	756	2.54	0.73	286	2.51	0.80	48	2.64	0.81	1,087	0.62	NFR = FR1, FR1 = FR2, NFR = FR2

*p < 0.05, **p < 0.01, ***p < 0.001.

**TABLE 3:** ANOVA comparisons among the three risk groups for mathematical difficulties (MD) for all variables.

	No family risk for MD (NFR)			One parent risk for MD (FR1)			Both parents risk for MD (FR2)					
	*N*	*M*	*SD*	*N*	*M*	*SD*	*N*	*M*	*SD*	*df* within groups	F	Pairwise comparisons (Bonferroni)
**Reading fluency (*z*-scores)**												
Grade 1	963	0.17	0.87	383	–0.11	0.78	63	–0.49	0.82	1,406	21.76***	NFR > FR1, FR1 > FR2, NFR > FR2
Grade 2	941	0.19	0.85	369	–0.14	0.78	62	–0.48	0.86	1,369	25.19***	NFR > FR1, FR1 > FR2, NFR > FR2
Grade 3	927	0.17	0.83	369	–0.09	0.81	60	–0.36	0.82	1,353	17.16***	NFR > FR1, FR1 = FR2, NFR > FR2
Grade 4	907	0.20	0.82	360	–0.14	0.81	58	–0.35	0.88	1,322	23.00***	NFR > FR1, FR1 = FR2, NFR > FR2
Grade 7	700	0.18	0.86	263	–0.10	0.83	32	–0.21	1.05	992	9.36***	NFR > FR1, FR1 = FR2, NFR > FR2
Grade 9	686	0.19	0.86	254	–0.05	0.84	32	–0.26	0.74	969	7.91***	NFR > FR1, FR1 = FR2, NFR > FR2
**Reading comprehension**												
Grade 1	961	6.06	3.13	379	5.13	3.19	63	4.22	3.31	1,400	19.46***	NFR > FR1, FR1 = FR2, NFR > FR2
Grade 2	928	9.03	2.47	367	8.23	2.74	61	6.75	3.13	1,353	31.35***	NFR > FR1, FR1 > FR2, NFR > FR2
Grade 3	925	9.42	2.03	368	8.90	2.13	60	8.47	2.48	1,350	12.59***	NFR > FR1, FR1 = FR2, NFR > FR2
Grade 4	906	8.64	2.29	360	7.78	2.62	58	7.62	2.25	1,321	19.54***	NFR > FR1, FR1 = FR2, NFR > FR2
Grade 7	696	7.06	2.56	263	6.42	2.53	32	5.53	2.24	988	10.27***	NFR > FR1, FR1 = FR2, NFR > FR2
Grade 9	681	7.45	2.36	251	6.84	2.45	32	6.09	2.37	961	9.87***	NFR > FR1, FR1 = FR2, NFR > FR2
**Arithmetic fluency**												
Grade 1	962	11.20	4.11	383	10.17	4.02	63	8.94	3.86	1,405	15.82***	NFR > FR1, FR1 = FR2, NFR > FR2
Grade 2	938	17.08	4.70	368	15.46	4.90	62	13.68	4.55	1,365	26.75***	NFR > FR1, FR1 > FR2, NFR > FR2
Grade 3	927	20.42	4.38	369	19.20	4.47	60	17.57	4.73	1,353	19.27***	NFR > FR1, FR1 > FR2, NFR > FR2
Grade 4	906	17.84	3.79	360	16.63	4.05	58	14.88	4.36	1,321	25.10***	NFR > FR1, FR1 > FR2, NFR > FR2
Grade 7	692	14.29	3.86	261	13.67	3.52	32	12.38	3.53	982	5.94**	NFR > FR1, FR1 = FR2, NFR > FR2
Grade 9	681	15.57	3.76	249	14.65	3.79	33	13.36	3.69	960	9.62***	NFR > FR1, FR1 = FR2, NFR > FR2
**Parental education**												
Mother	990	4.48	1.49	403	3.85	1.35	72	4.25	3.95	1,462	50.71***	NFR > FR1, FR1 < FR2, NFR = FR2
Father	749	4.35	1.51	292	3.76	1.40	51	3.20	1.17	1,089	27.52***	NFR > FR1, FR1 > FR2, NFR > FR2
**Home learning environment factors (mean composites)**												
Shared reading, mother	988	2.94	1.13	401	2.92	1.18	74	2.72	1.05	1,460	1.29	NFR = FR1, FR1 = FR2, NFR = FR2
Shared reading, father	738	2.39	1.15	287	2.30	1.18	52	2.12	1.18	1,074	1.86	NFR = FR1, FR1 = FR2, NFR = FR2
Teaching, mother	991	2.60	0.81	405	2.60	0.94	74	2.60	0.85	1,467	0.09	NFR = FR1, FR1 = FR2, NFR = FR2
Teaching, father	745	2.54	0.75	291	2.60	0.74	52	2.21	0.77	1,085	5.67**	NFR = FR1, FR1 > FR2, NFR > FR2

*p < 0.05, **p < 0.01, ***p < 0.001.

To evaluate model fit, chi-square values and a set of fit indexes were used as follows: (a) CFI; (b) RMSEA, and (c) SRMR. Good model fit is indicated by a small, preferably non-significant χ^2^, CFI > 0.95, RMSEA < 0.06, and SRMR < 0.08 (Hu and Bentler, 1999). Because the chi-square test is sensitive to a large sample size, the chi-square statistics were not regarded as conclusive.

## Results

### Descriptive Statistics

Descriptive statistics for children’s skill development and HLE measures are reported for all participants in Table 1, as a function of FR for reading difficulties in Table 2, and as a function of FR for mathematical difficulties in Table 3. One-way ANOVAs were conducted to compare the children with no FR (NFR), the children with one parent with difficulties (FR1), and the children with two parents with difficulties (FR2) (Tables 2, 3) and showed significant differences between the NFR group, FR1 group, and FR2 group for all the skills throughout Grades 1–9 except arithmetic skills in Grade 7 as a function of parental reading difficulties. This analysis also demonstrated that parental education was significantly higher in the NFR group than in the FR1 and FR2 groups, whereas there were no group differences in the home environment measures.

Pairwise comparisons of the groups with parental reading difficulties (FR1 and FR2) revealed significant differences in children’s reading fluency in Grades 1 and 4 (Table 2), whereas comparisons of the groups with parental mathematical difficulties (FR1 and FR2) showed that children significantly differed in their reading comprehension skills in Grades 1 and 2 as well as in arithmetical fluency skills in Grades 2, 3, and 4 (Table 3).

Pearson correlation coefficients are reported across all measures in Table 4. All skills were significantly related with one another, but the strongest correlations were found in lower grades. The correlations between the reading and mathematical measures and the home teaching environment and shared reading were small, ranging from 0.01 to 0.19.

**TABLE 4:** Correlations between all variables.

	1	2	3	4	5	6	7	8	9	10	11	12	13	14
1. Grade 1	1													
2. Grade 2	0.80**	1												
3. Grade 3	0.75**	0.82**	1											
4. Grade 4	0.71**	0.79**	0.85**	1										
5. Grade 7	0.61**	0.67**	0.71**	0.75**	1									
6. Grade 9	0.58**	0.64**	0.67**	0.72**	0.81**	1								
7. Grade 1	0.63**	0.60**	0.55**	0.56**	0.47**	0.45**	1							
8. Grade 2	0.48**	0.49**	0.47**	0.47**	0.42**	0.43**	0.53**	1						
9. Grade 3	0.33**	0.35**	0.34**	0.37**	0.33**	0.35**	0.39**	0.48**	1					
10. Grade 4	0.37**	0.39**	0.40**	0.41**	0.41**	0.39**	0.44**	0.55**	0.47**	1				
11. Grade 7	0.26**	0.30**	0.26**	0.30**	0.37**	0.39**	0.36**	0.45**	0.40**	0.51**	1			
12. Grade 9	0.29**	0.32**	0.28**	0.30**	0.35**	0.40**	0.37**	0.43**	0.36**	0.43**	0.51**	1		
**Arithmetic Fluency (z-scores)**														
13. Grade 1	0.51**	0.48**	0.46**	0.46**	0.33**	0.32**	0.40**	0.29**	0.19**	0.21**	0.14**	0.17**	1	
14. Grade 2	0.47**	0.50**	0.49**	0.49**	0.39**	0.37**	0.39**	0.32**	0.23**	0.27**	0.19**	0.16**	0.69**	1
15. Grade 3	0.46**	0.49**	0.53**	0.53**	0.40**	0.38**	0.40**	0.32**	0.25**	0.27**	0.20**	0.17**	0.64**	0.75**
16. Grade 4	0.44**	0.48**	0.50**	0.53**	0.41**	0.40**	0.40**	0.34**	0.27**	0.33**	0.24**	0.20**	0.61**	0.70**
17. Grade 7	0.36**	0.37**	0.37**	0.37**	0.41**	0.41**	0.33**	0.32**	0.27**	0.31**	0.34**	0.29**	0.51**	0.59**
18. Grade 9	0.37**	0.37**	0.34**	0.36**	0.39**	0.40**	0.35**	0.32**	0.27**	0.29**	0.35**	0.31**	0.54**	0.59**
**Parental Reading Difficulties**														
19. Mother	−0.13**	−0.16**	−0.12**	−0.14**	−0.10**	−0.08*	−0.12**	−0.12**	−0.10**	−0.13**	–0.02	–0.05	−0.06*	−0.07**
20. Father	−0.16**	−0.18**	−0.14**	−0.15**	−0.13**	−0.12**	−0.13**	−0.12**	−0.10**	−0.08**	−0.12**	−0.10**	−0.09**	−0.10**
21. Mother	−0.12**	−0.13**	−0.11**	−0.13**	−0.09**	−0.07*	−0.10**	−0.15**	−0.11**	−0.14**	−0.10**	−0.09**	−0.10**	−0.15**
22. Father	−0.14**	−0.15**	−0.13**	−0.14**	−0.11**	−0.11**	−0.15**	−0.15**	−0.10**	−0.11**	−0.12**	−0.11**	−0.14**	−0.14**
**Parental Education**														
23. Mother	0.13**	0.15**	0.12**	0.16**	0.13**	0.17**	0.18**	0.17**	0.17**	0.22**	0.19**	0.19**	0.12**	0.15**
24. Father	0.13**	0.16**	0.12**	0.16**	0.14**	0.13**	0.18**	0.18**	0.19**	0.20**	0.17**	0.18**	0.12**	0.16**
**Home Learning Environment**														
25. Shared reading, mother	0.02	0.05*	0.04	0.07*	0.07*	0.09**	0.10**	0.14**	0.11**	0.20**	0.19**	0.17**	–0.01	0.01
26. Shared reading, father	0.02	0.04	0.06	0.09**	0.08*	0.08*	0.11**	0.12**	0.16**	0.19**	0.14**	0.15**	0.01	0.05
27. Teaching literacy, mother	0.08**	0.07**	0.10**	0.06*	0.08*	0.09**	0.10**	0.06*	0.03	0.07**	0.03	0.06	0.02	0.03
28. Teaching literacy, father	0.02	–0.02	–0.01	–0.01	0.04	0.03	0.06	0.01	0.02	0.01	0.03	0.06	0.01	–0.01
29. Teaching numeracy, mother	–0.04	−0.05*	–0.01	–0.03	–0.04	–0.02	–0.03	–0.05	–0.05	–0.00	–0.05	–0.03	0.00	0.02
30. Teaching numeracy, father	–0.06	–0.05	–0.04	–0.05	–0.02	–0.00	–0.02	–0.03	–0.01	0.01	0.01	–0.00	0.05	0.04

	**15**	**16**	**17**	**18**	**19**	**20**	**21**	**22**	**23**	**24**	**25**	**26**	**27**	**28**	**29**	**30**

1. Grade 1																
2. Grade 2																
3. Grade 3																
4. Grade 4																
5. Grade 7																
6. Grade 9																
7. Grade 1																
8. Grade 2																
9. Grade 3																
10. Grade 4																
11. Grade 7																
12. Grade 9																
**Arithmetic Fluency (z-scores)**																
13. Grade 1																
14. Grade 2																
15. Grade 3	1															
16. Grade 4	0.77**	1														
17. Grade 7	0.60**	0.68**	1													
18. Grade 9	0.61**	0.67**	0.75**	1												
**Parental Reading Difficulties**																
19. Mother	−0.07**	–0.05	–0.02	–0.06	1											
20. Father	−0.09**	−0.10**	−0.09**	−0.08**	–0.04	−0.08*	0.10**	1								
21. Mother	−0.13**	−0.14**	−0.07*	−0.09**	0.30**	0.13**	1									
22. Father	−0.13**	−0.17**	−0.10**	−0.15**	0.16**	0.38**	0.13**	1								
**Parental Education**																
23. Mother	0.17**	0.18**	0.21**	0.19**	−0.15**	−0.10**	−0.23**	−0.15**	1							
24. Father	0.15**	0.20**	0.16**	0.19**	−0.06*	−0.14**	−0.14**	−0.20**	0.53**	1						
**Home Learning Environment**																
25. Shared reading, mother	–0.01	0.01	–0.01	0.05	−0.07**	–0.02	–0.05	0.01	0.21**	0.12**	1					
26. Shared reading, father	0.05	0.06	0.05	0.09*	–0.02	–0.03	–0.06	–0.03	0.23**	0.20**	0.48**	1				
27. Teaching literacy, mother	0.03	–0.00	–0.01	–0.04	–0.04	–0.03	–0.04	0.03	−0.06*	–0.05	0.14**	0.04	1			
28. Teaching literacy, father	–0.02	–0.04	0.00	0.05	0.08**	–0.04	0.03	–0.06	0.00	–0.01	0.12**	0.24**	0.26**	1		
29. Teaching numeracy, mother	0.02	0.01	0.00	–0.02	–0.02	0.01	–0.02	0.00	−0.11**	−0.10**	0.12**	0.01	0.68***	0.20**	1	
30. Teaching numeracy, father	0.02	0.01	0.06	0.03	0.01	–0.02	0.00	−0.10**	0.03	0.00	0.07*	0.19**	0.19**	0.67***	0.22**	1

*p < 0.05, **p < 0.01, *** p < 0.001.

### The Model for Reading Fluency

**FIGURE 2:**
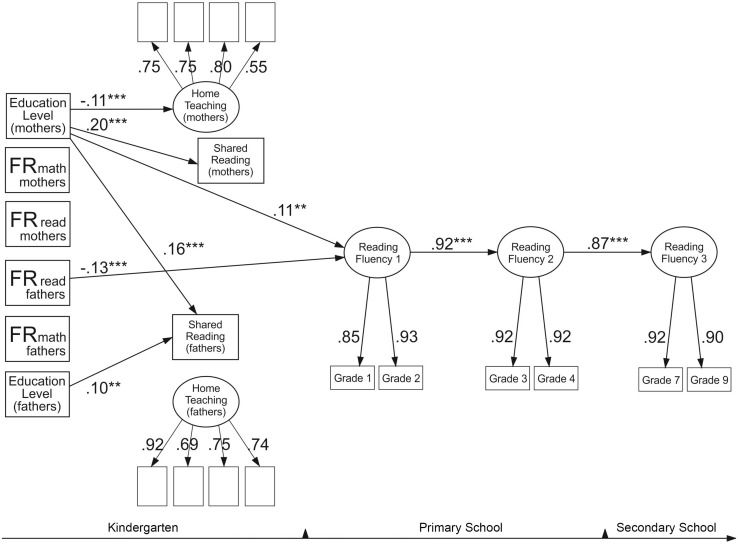
Reading fluency model. The model shows only significant standardized paths. Familial risk (FR for reading and mathematical difficulties of mothers and fathers). *p < 0.05, **p < 0.01, ***p < 0.001. Two significant residual correlations were included in the model: between the mothers’ and fathers’ teaching factor (0.27**) and between the mothers’ and fathers’ shared reading variables (0.47**).

Figure 2 presents the final model for reading fluency with statistically significant standardized estimates, and Table 5 reports all the path estimates and residual correlations of the model. The model fitted the data well: χ^2^(171) = 247.90, *p* < 0.001, RMSEA = 0.02, CFI = 0.98, SRMR = 0.03. Two significant predictors of reading fluency emerged: children’s reading fluency at the first time point was predicted by fathers’ reading difficulties and by mothers’ educational level. That is, fathers’ reading difficulties and lower maternal education predicted poorer performance in reading fluency tasks among their children. However, the effects were small, explaining 2 and 1% of the variance, respectively. There were no significant effects of any of the home environment factors on reading fluency and parental reading, and mathematical difficulties did not predict the home environment factors. However, higher levels of education among mothers predicted less time spent on teaching activities and more time spent on shared reading. In addition, higher levels of education of mothers and fathers were associated with more shared reading with fathers. Again, the amounts of explained variance in the home environment owing to educational level were low, between 1 and 4%. This model did not reveal any significant indirect effects. Reading fluency demonstrated very high stability across time. The first time point explained 85% of the variance in reading fluency at the second time point, which then explained 75% of the variance at the third time point.

**TABLE 5:** All regression paths and residual correlations in the three models.

Path estimates	Model for reading fluency: estimate (s.e.)	Model for reading comprehension (s.e.): estimate (s.e.)	Model for arithmetic fluency: estimate (s.e.)
FR for reading, mothers → home teaching, mothers	−0.05 (0.03)	−0.05 (0.03)	−0.05 (0.03)
FR for reading, mothers → shared reading, mothers	−0.04 (0.03)	−0.04 (0.03)	−0.04 (0.03)
FR for math, mothers → home teaching, mothers	−0.06 (0.05)	−0.06 (0.05)	−0.06 (0.05)
FR for math, mothers → shared reading, mothers	0.02 (0.03)	0.02 (0.03)	0.02 (0.03)
FR for reading, mothers→ skills at Time Point 1	−0.05 (0.03)	−0.06 (0.04)	−0.00 (0.03)
FR for math, mothers→ skills at Time Point 1	−0.06 (0.03)	−0.10* (0.04)	−0.11** (0.03)
Education level, mothers → skills at Time Point 1	0.11** (0.04)	0.13** (0.04)	0.06 (0.04)
Education level, mothers → skills at Time Point 2			0.09*** (0.02)
Education level, mothers → home teaching, mothers	−0.11*** (0.03)	−0.11*** (0.03)	−0.11*** (0.03)
Education level, mothers → shared reading, mothers	0.20*** (0.03)	0.20*** (0.03)	0.20*** (0.03)
Shared reading, mothers → skills at Time Point 1	−0.01 (0.04)	0.05 (0.04)	−0.01 (0.04)
Shared reading, mothers → skills at Time Point 2		0.13*** (0.03)	
At-home teaching, mother → skills at Time Point 1	−0.02 (0.04)	0.02 (0.04)	−0.01 (0.04)
FR for reading, fathers → home teaching, fathers	0.01 (0.03)	0.01 (0.03)	0.01 (0.03)
FR for reading, fathers → shared reading, fathers	0.01 (0.03)	0.01 (0.03)	0.01 (0.03)
FR for math, fathers → home teaching, fathers	−0.07 (0.04)	−0.07 (0.04)	−0.07 (0.04)
FR for math, fathers → shared reading, fathers	−0.01 (0.03)	−-0.01 (0.03)	−0.01 (0.03)
FR for reading, fathers → skills at Time Point 1	−0.13*** (0.04)	−0.07 (0.04)	−0.04 (0.04)
FR for math, fathers → skills at Time Point 1	−0.05 (0.04)	−0.10* (0.04)	−0.11** (0.04)
Education level, fathers → skills at Time Point 1	0.06 (0.04)	0.14** (0.04)	0.10** (0.04)
Education level, fathers → home teaching, fathers	−0.01 (0.03)	−0.01 (0.03)	−0.01 (0.03)
Education level, fathers → shared reading, fathers	0.10** (0.03)	0.10** (0.03)	0.10** (0.03)
Shared reading, fathers → skills at Time Point 1	0.02 (0.04)	0.10* (0.04)	0.01 (0.04)
At-home teaching, fathers → skills at Time Point 1	−0.04 (0.04)	−0.02 (0.04)	0.00 (0.03)
Skills at Time Point 1 → Skills at Time Point 2	0.92*** (0.01)	0.85*** (0.03)	0.90*** (0.01)
Skills at Time Point 2 → Skills at Time Point 3	0.87*** (0.02)	0.93*** (0.03)	0.88*** (0.02)
Education level, mothers → Shared reading, fathers	0.16*** (0.03)	0.16*** (0.03)	0.16*** (0.03)
**Residual covariances**			
Home teaching, mothers with home teaching, fathers	0.25*** (0.04)	0.25*** (0.04)	0.25*** (0.04)
Shared reading, mothers with home teaching, mothers	0.15***(0.03)	0.15*** (0.03)	0.15*** (0.03)
Shared reading, mothers with home teaching, fathers	0.13*** (0.03)	0.13*** (0.03)	0.13*** (0.03)
Shared reading, fathers with home teaching, fathers	0.26*** (0.03)	0.26*** (0.03)	0.26*** (0.03)
Shared reading, fathers with shared reading, mothers	0.44*** (0.03)	0.44*** (0.03)	0.44*** (0.03)

*p < 0.05, **p < 0.01, ***p < 0.001. Some regression and correlation paths were not initially hypothesized but were later added based on the modification indices.

### The Model for Reading Comprehension

**FIGURE 3:**
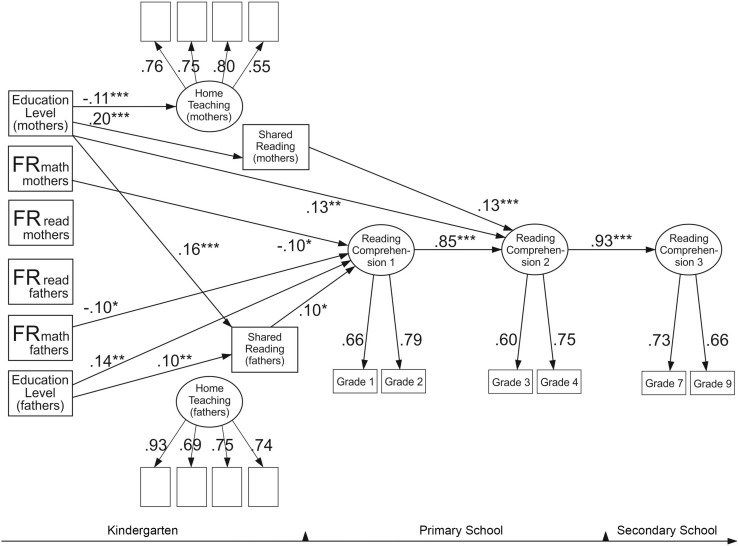
Reading comprehension model. The model shows only significant standardized paths. *p < 0.05, **p < 0.01, ***p < 0.001. Familial risk (FR for reading and mathematical difficulties of mothers and fathers). Two significant residual correlations were included in the model: between the mother’s and father’s teaching factor (0.27**) and between the shared reading variables (0.47**).

Figure 3 reports the final model for reading comprehension. The model fitted the data well: χ^2^(170) = 248.42, *p* < 0.001, RMSEA = 0.02, CFI = 0.97, SRMR = 0.03. The model suggested several statistically significant predictors of reading comprehension. Mothers’ and fathers’ mathematical difficulties predicted poorer reading comprehension among children, each predicting 1% of the variance. Mothers’ and fathers’ levels of education were significant positive predictors of children’s reading comprehension, each explaining 2% of the variance. Shared reading with fathers was also found to have a direct positive effect on children’s reading comprehension (explaining 1% of the variance) at the first time point, whereas shared reading with mothers was predictive of children’s comprehension at the second time point (explaining 2% of the variance). In addition, higher levels of education among mothers predicted more time spent on shared reading and less time spent on teaching activities. The higher levels of education of mothers and fathers were associated with more shared reading with fathers. This model did not reveal any significant indirect effects. In addition, reading comprehension demonstrated very high stability across time. The first time point explained 72% of the variance in reading comprehension at the second time point, which then explained 87% of the variance at the third time point.

### The Model for Arithmetic Fluency

**FIGURE 4:**
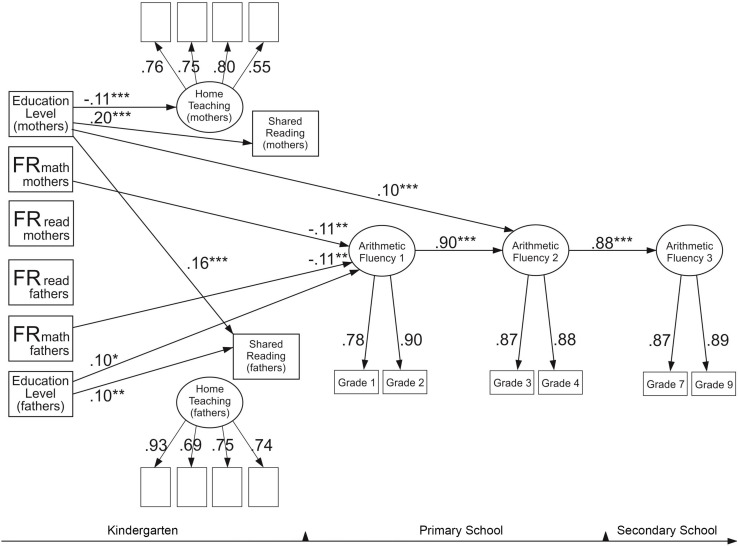
Arithmetic fluency model. The model shows only significant standardized paths. *p < 0.05, **p < 0.01, ***p < 0.001. FR, familial risk (FR for reading and mathematical difficulties of mothers and fathers). Two significant residual correlations were included in the model: between the mother’s and father’s teaching factor (0.27**) and between the shared reading variables (0.47**).

Figure 4 reports the model for arithmetic fluency. The model fitted the data well: χ^2^(170) = 255.33, *p* < 0.001, RMSEA = 0.02, CFI = 0.979, SRMR = 0.03. Similarly to the comprehension model, this model revealed that only mathematical but not reading difficulties of mothers and fathers predicted children’s mathematical skills, each explaining 1% of the variance. Mothers’ and fathers’ levels of education were also significant predictors of children’s arithmetic fluency, with fathers’ education explaining 1% of the variance at the first time point and mothers’ education explaining 1% of the variance at the second time point. No significant effects of any home environment factors for predicting children’s arithmetic fluency were observed. Higher levels of education among mothers predicted less time spent on teaching activities and more time spent on shared reading. Higher levels of education among mothers and fathers predicted more shared reading with fathers. This model did not reveal any significant indirect associations. Similarly to reading skills, arithmetic fluency demonstrated very high stability across time. The first time point explained 81% of the variance in mathematics skills at the second time point, which then explained 77% of the variance at the third time point.

## Discussion

In this study, our main goal was to gain more understanding of the basis of reading and mathematical comorbidity by examining the transmission of parental reading and mathematical difficulties (FR) onto children’s reading and mathematical skills. We examined both direct effects of FR on children’s skill development and indirect effects of FR via formal and informal home learning activities. To provide insights into the underpinning processes of the frequently occurring comorbidity of reading and mathematical difficulties, our analysis included mathematical and reading skills, FR for reading and mathematical difficulties coming from both parents, as well as home environment measures for both literacy and numeracy activities. Parental educational level was included as a control measure. Our findings indicated the direct effects of FR on children’s skills but no indirect effects via the home environment. Indeed, neither mathematical nor reading difficulties of the parents predicted the frequency of shared reading and parental teaching activities. Higher levels of parental education, on the contrary, predicted more frequent shared reading with both parents and less frequent teaching activities with mothers. In addition, we found that parental mathematical difficulties predicted not only children’s mathematical skills but also their reading comprehension, whereas parental reading difficulties predicted only children’s reading fluency. This suggests that the mathematical difficulties of parents increase their children’s liability for developing not only mathematical difficulties but also reading comprehension difficulties. Finally, of the home environment measures, shared reading predicted reading comprehension in Grades 1 and 2 as well as faster development of comprehension skills from Grades 1 and 2 to Grades 3 and 4, whereas more literacy and numeracy teaching activities did not predict skills. These findings suggest that children’s learning difficulties arise from a complex interaction of multiple risk factors (inherited deficits and environmental influences).

### Familial Risk as a Predictor of Reading and Mathematical Skills

The results suggested significant within-domain effects of parental skills on children’s skills, particularly for parental mathematical difficulties. Both mothers’ and fathers’ mathematical difficulties predicted poorer performance in arithmetic fluency among their children. Furthermore, fathers’ reading difficulties predicted their children’s reading fluency. Mothers’ reading difficulties, however, were not predictive of any of the children’s skills. These findings are consistent with those of previous studies showing significant FR effects for mathematics (Shalev and Gross-Tsur, 2001; Soares et al., 2018) and reading (Elbro et al., 1998; Torppa et al., 2011, 2015; van Bergen et al., 2014a; Hulme et al., 2015; Esmaeeli et al., 2019). However, the effect sizes were modest, with FR (coming from each parent) predicting approximately 1% of children’s skills in Grades 1 and 2. Nevertheless, this effect size is comparable to that in earlier studies in which FR was self-reported and not tested. Recently, Esmaeeli et al. (2019) reported that in their study, FR explained 3% of the variance in children’s reading skills. However, Torppa et al. (2011) and van Bergen et al. (2014a) estimated that 8–16% and 11% of children’s reading skills, respectively, can be predicted by FR when it is identified with parental skill assessments. Undoubtedly, parental testing is a more reliable measure to detect FR than self-reports, although the correlation between formally tested reading skills and self-reported difficulties has been reported to be as high as 0.80 (van Bergen et al., 2014a).

In line with the previous FR studies, the results of our models revealed significant differences in children’s skills between groups with and without FR. For some skill measures, the results further suggested a stepwise pattern wherein the group with one parent FR had stronger skills than the group with FR owing to two parents. This evidence suggests that the dual parent learning difficulty constitutes an aggravated risk for children’s skill development. This finding is in line with the MDM and fits with the suggestions of the continuous liability distribution of FR (Snowling et al., 2003; Pennington, 2006; van Bergen et al., 2012). The pattern was present for parental mathematical difficulties in four arithmetic assessments, two reading fluency assessments, and one reading comprehension assessment. However, for parental reading difficulties, the pattern was present only for the reading fluency of children in Grades 1 and 4.

Significant cross-domain effects of FR on children’s skills were also identified but only for parental mathematical difficulties. Both mothers’ and fathers’ mathematical difficulties predicted children’s reading comprehension but not reading fluency. Moreover, children’s mathematical skills did not appear to be associated with FR for reading difficulties. These paths from FR to mathematical difficulties lend support to the argument that reading and mathematical difficulties have both common and distinct underpinnings (Landerl and Moll, 2010; Carvalho and Haase, 2019) and point to an intergenerational transmission of multiple deficits, as posited by Pennington’s MDM. The findings support those of earlier studies indicating that mathematical difficulties more often co-occur with reading difficulties than the other way around (Landerl and Moll, 2010; Carvalho and Haase, 2019). The findings do not, however, explain the comorbidity of reading and mathematical difficulties that is often found using fluency-based assessments (Moll et al., 2019). The processes underlying the specific link between children’s reading comprehension and parental mathematical difficulties need to be examined further. Some research has indicated that the genetic correlations of mathematical skills with reading comprehension are significantly higher than those with decoding (Harlaar et al., 2012). Furthermore, a strong association has been found between children’s reading comprehension and mathematical reasoning (Pimperton and Nation, 2010), which may in part explain why we found parental mathematical difficulties predicting children’s reading comprehension.

### Home Learning Environment as a Predictor of Children’s Reading and Arithmetic Skills

At-home teaching activities seemed to have neither direct nor indirect effects on children’s skills, which stands in contrast with our hypothesis and earlier research (Martini and Sénéchal, 2012; Sénéchal and Lefevre, 2014; Skwarchuk et al., 2014; Sénéchal, 2015; Puglisi et al., 2017; Napoli and Purpura, 2018). Our findings are in line with some other research (Missall et al., 2015; Zippert and Rittle-Johnson, 2020) and could be viewed as supportive evidence for the argument that gains from formal home activities tend to be negligibly small and short-term in the context of transparent languages and fade away once children enter school (Manolitsis et al., 2013; Silinskas et al., 2020). Indeed, highly regular orthographies speed up the process of reading acquisition allowing children to reach good reading levels with the support of high-quality phonics teaching at school (Aro, 2017), which explains why providing early reading instruction at home does not ensure any long-term advantage. It is also important to stress that Finland has succeeded in promoting educational equality by creating a welfare state, which provides early educational support in schools to every child reducing the need for home teaching and the extent to which a family’s socioeconomic background affects their child’s development (e.g., Reinikainen, 2012).

At the same time, as expected, shared reading organized by both mothers and fathers had significant direct effects on children’s reading comprehension in lower grades, which is in line with earlier findings pointing to the influence of informal literacy inputs on beginners’ reading comprehension (Foy and Mann, 2003; Sénéchal, 2006, 2015; Torppa et al., 2007; Martini and Sénéchal, 2012; Manolitsis et al., 2013; Sénéchal and Lefevre, 2014; Hamilton et al., 2016; Puglisi et al., 2017). However, no effects of shared reading were found for arithmetic or reading fluency, which is consistent with the findings of earlier studies that investigated the effects of informal meaning-related home activities on children’s decoding skills, symbolic number knowledge, and non-symbolic arithmetic skills (Sénéchal et al., 2008; Martini and Sénéchal, 2012; Sénéchal and Lefevre, 2014; Napoli and Purpura, 2018; Esmaeeli et al., 2019). The reason for reading comprehension being associated with shared reading is typically explained by its impact on oral language (Torppa et al., 2007; Sénéchal et al., 2008; Martini and Sénéchal, 2012; Sénéchal and Lefevre, 2014; Hamilton et al., 2016; Silinskas et al., 2020). Similar to the predictive effects of FR, the effects of shared reading on children’s comprehension were rather small—less than 2%. The modest variance explained by informal learning likely stems from the same reasons listed above in regards to the predictive role of formal activities at home. In addition, Puglisi et al. (2017) reported that the relationship between informal literacy learning activities and children’s skills is mostly accounted for by parental skills and might reflect a gene-environment correlation. Interestingly, however, this study found that shared reading with mothers was predictive of the reading comprehension of children in Grades 3 and 4 even with the inclusion of FR, as well as over and above the autoregressor, suggesting that the improvement in reading comprehension during the early school years was partially predicted by shared reading.

### Familial Risk and the Home Learning Environment

The models indicated that FR for neither reading nor mathematical difficulties predicted at-home teaching or shared reading—parents with difficulties read with their children and taught academic skills in the same way as the parents without difficulties. This is in line with previous research (Elbro et al., 1998; Laakso et al., 1999; Torppa et al., 2007; Hamilton et al., 2016) suggesting that parental reading and mathematical difficulties are not transmitted to their children via the home environment. Intriguingly, higher levels of education among mothers predicted significantly less time spent on teaching activities and more time spent on shared reading. In other words, FR predicted neither formal nor informal home environment activities whereas maternal education predicted both. In the more educated homes, fathers also spent more time reading with their children. It is possible that parents with lower levels of education are more inclined to expect their children’s possible school failure or, alternatively, that they increase the volume of home teaching activities when their children display early signs of difficulties (Blevins-Knabe and Musun-Miller, 1996; Silinskas et al., 2010; Sénéchal and Lefevre, 2014).

In addition, and contrary to our hypothesis, we did not find FR having a significant indirect effect on children’s skills via the home environment. This negative finding is in line with Esmaeeli et al. (2019), who despite their hypothesis also failed to find significant indirect paths from FR for reading difficulties to children’s skill. That said, however, it is important to not completely discard the influence of FR on the home environment. Indeed, Esmaeeli et al. (2018) made a reasonable argument that FR might be negatively affecting the home environment both directly and indirectly through parental education because the FR status is likely to be a contributing factor to lower parental education, as was previously reported both in Finland and in other countries (McLaughlin et al., 2014; Aro et al., 2019). Interestingly, some studies (Scarborough et al., 1991; Bus et al., 1995; Elbro et al., 1998; Snowling, 2000; Leinonen et al., 2001; Torppa et al., 2007) showed that parents with learning difficulties read less than their control counterparts and thus may provide less positive parental models.

### Limitations and Future Research

The present study has limitations in regard to the measures employed. First, similarly to previous investigations (e.g., Silinskas et al., 2010; Esmaeeli et al., 2018, 2019), this study deployed parental self-reports of HLE and HNE, which are liable to social desirability bias. Moreover, the measures mostly focused on assessing the formal activities of the home environment and had only one question assessing informal HLE and no questions tapping into informal HNE. Therefore, an important goal for future research is to incorporate a wider range of assessment measures for HLE and HNE which, in combination with longitudinal study designs, render an essentially more reliable prediction than cross-sectional studies alone. However, even well-founded longitudinal associations are far from being interpreted causally. Thus, randomized controlled trials testing various HLE and HNE interventions are needed to aid in the understanding of causal effects. Second, the quality of at-home learning can vary significantly and could be an additional predictor (Siraj-Blatchford, 2010; Kluczniok et al., 2013). The lack of measures capturing the quality of home teaching could be one of the reasons behind the small amount of variance explained by the home environment activities, and future studies should take this into account. Third, future research would benefit from using a more comprehensive assessment of the FR status. The self-report measure for parents used in the present study was short and simple. Nevertheless, this study revealed significant FR effects on children’s reading and mathematical skills that are comparable to those found in previous FR studies (Silinskas et al., 2010; Esmaeeli et al., 2018, 2019).

In this study, we were particularly interested in arithmetic fluency as it starts to develop in early grades and forms the foundation not only for more complex arithmetic skills (Carr and Alexeev, 2011) but also for mathematical reasoning (Powell et al., 2016). The defining feature of specific mathematical difficulty in the primary grades is a poorly developed subtraction and addition fluency (e.g., Jordan et al., 2003). However, a desirable goal is making the mathematical assessment more comprehensive by including, for example, a mathematical reasoning measure. The link between reading comprehension and mathematical reasoning has been previously reported (Pimperton and Nation, 2010) suggesting that the possible intergenerational connection of these skills could to be another avenue for future research. Finally, it is important to assess not only the quantity but also the quality of home learning activities, which represents a serious challenge but could be achieved in future research with the use of qualitative case studies (Siraj-Blatchford, 2010).

## Conclusion

**FIGURE 5:**
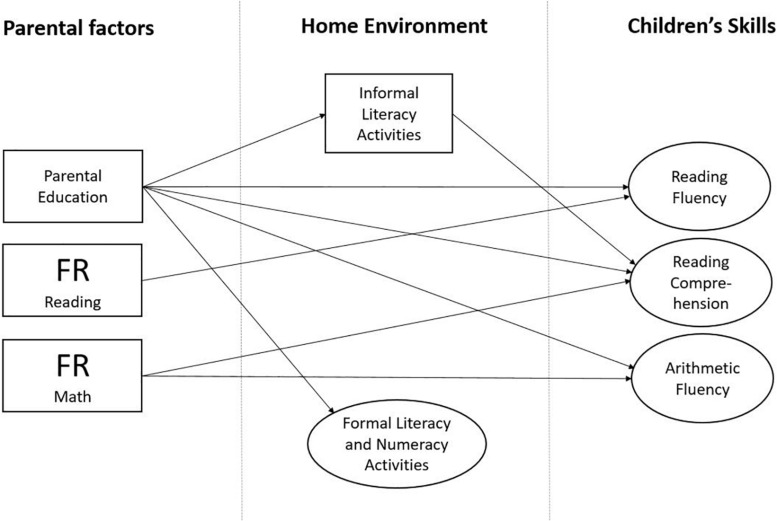
Visual summary. The figure shows all significant paths found in this study.

We have summarized visually the results of this study in Figure 5. The key finding is that FR for both reading and mathematical difficulties had direct effects on children’s skills—the difference between groups with and without FR became apparent in the early grades and remained stable till the last time point of assessment in Grade 9. More specifically, FR for mathematical difficulties predicted both mathematical and reading comprehension difficulties in children, whereas FR for reading difficulties was predictive of children’s reading fluency difficulties only. However, there were no indirect effects of FR via the home environment. Moreover, we failed to detect any effect of the FR status on the home environment. Another important finding is that shared reading was the only component of the home environment that predicted faster development of children’s skills: more specifically, the reading comprehension in Grades 3 and 4. At the same time, more educated mothers and fathers spent more time reading with their children, whereas mothers with lower levels of education were more likely to focus on at-home teaching. These findings might appear somewhat counterintuitive and therefore call for more nuanced research of learning milieus at home. In particular, more attention needs to be paid on how to support the home learning activities of academically under-privileged parents who are trying their best to give their children a head start.

## Data Availability Statement

The raw data supporting the conclusions of this article will be made available by the authors, without undue reservation, to any qualified researcher.

## Ethics Statement

The studies involving human participants were reviewed and approved by the Ethical Committee of the University of Jyväskylä in 2006. Written informed consent to participate in this study was provided by the participants’ legal guardian/next of kin.

## Author Contributions

DK drafted the first version of the current manuscript. MP, MT, and DK contributed to the data analysis. GS, M-KL, PN, A-MP, and MT were responsible for the data collection and commented on the manuscript. All authors contributed to the manuscript drafting, and read and approved the submitted version.

## Conflict of Interest

The authors declare that the research was conducted in the absence of any commercial or financial relationships that could be construed as a potential conflict of interest.
